# Effects of Subjective Socioeconomic and Social Statuses on Food Choice in Adolescents

**DOI:** 10.1177/13591053261442340

**Published:** 2026-05-07

**Authors:** Jenna R. Cummings, Aimee E. Pink, Meegan R. Smith, Julia M. P. Bittner, Albert Lee, Bobby Cheon

**Affiliations:** a Department of Psychology, Institute of Population Health, University of Liverpool, Eleanor Rathbone Building, Bedford Street South, Liverpool, L69 7ZA, United Kingdom; b Institute of High Performance Computing (IHPC), Agency for Science, Technology and Research (A*STAR), 1 Fusionopolis Way, #16-16 Connexis, Singapore 138632, Republic of Singapore; c Institute for Human Development and Potential (IHDP), Agency for Science, Technology and Research (A*STAR), 30 Medical Drive, Brenner Centre for Molecular Medicine, Singapore, 117609, Republic of Singapore; d Social and Behavioral Sciences Branch, Division of Population Health Research, *Eunice Kennedy Shriver* National Institute of Child Health and Human Development, 6710B Rockledge Drive, Bethesda, MD, 20817, United States of America; e Division of Psychology, Nanyang Technological University, HSS-04-09, 14 Nanyang Drive, Singapore, 637332, Republic of Singapore

**Keywords:** adolescents, experiments, food choice, subjective social status, subjective socioeconomic status

## Abstract

Subjective status—the perception of one’s place in a socioeconomic or social structure—declines during adolescence, a developmentally sensitive period when low subjective status may be a fundamental threat. Although low subjective status manipulations during adulthood have triggered unhealthy food choices, causal effects on food choices during adolescence are unclear. In two preregistered online experiments, adolescents aged 14-17 years compared their families to those with higher socioeconomic statuses (*n* = 473) and themselves to those with higher social statuses in their schools (*n* = 775) and completed a food choice task. There were inconsistent effects of the subjective status manipulations on subjective statuses, and no statistically significant effects on food choices, in adolescents. Longitudinal studies examining fluctuations in subjective socioeconomic and social statuses and food choices across the school years, or experiments with alternative manipulations, are needed to examine potential influences of subjective statuses on food choices in adolescents.

## Introduction

There are persistent differences in food intake patterns of youth based on their socioeconomic statuses, that is, where their families are positioned in a socioeconomic structure. For example, in the last two decades, American youth with household income < 1.30 times the poverty level ate fewer total vegetables, nuts, and seeds compared to those with greater household income (Liu et al., 2020). American youth with parental education less than high school ate fewer dark-green vegetables, nuts, and seeds, and more processed meat, compared to those with greater parental education (Liu et al., 2020). Food accessibility majorly contributes to socioeconomic disparities in food choices (Larson et al., 2009). However, given that there is variability in food choices between individuals even when they have the same food access (Larson et al., 2009), it is important to understand how psychological aspects of socioeconomic status may furthermore influence food choices in youth.

Subjective socioeconomic status (SSES) is the *perception* of one’s place in the socioeconomic structure (Adler et al., 2000). Correlations between SSES and measures of objective socioeconomic status like household income are moderate (Quon and McGrath, 2014); for instance, when individuals have low household income, *some individuals will perceive themselves as low in socioeconomic standing* while others will perceive themselves as in the middle or high in it. SSES, independently from objective socioeconomic status, predicts behaviour (Quon and McGrath, 2014). The development of a perception of having few social and material resources may therefore be a psychological aspect of socioeconomic status that independently affects food choices in adolescents.

Adolescence is a key period for SSES to change because it is a time when individuals begin establishing independence from their parents in the broader socioeconomic structure (Goodman et al., 2001). For example, youth may start their first jobs or enter their first romantic relationships. Distinct neurobiological changes occur during this period too, with limbic systems underlying emotional experiences maturing far faster than prefrontal systems that underly decision making (Casey et al., 2008). These neurobiological changes make adolescents sensitive to social rewards and punishment and, thus, especially alert to social comparison (Van der Aar et al., 2018). Indeed, although children versus adults tend to show upward bias in SSES—having higher SSES than would be expected based on their objective socioeconomic status—adolescents ≥ 14-years-old do not have this upward bias (Goodman et al., 2001). Moreover, in children reporting low SSES at age 12 years, SSES tends to get even lower during adolescence and emerging adulthood (Goodman et al., 2015).

In addition to SSES, researchers have explored subjective social statuses (SSS) in young people—perceptions of one’s standing in their local community (here, school) *based on social factors like respect, popularity, and achievement* rather than socioeconomic factors like household income (Goodman et al., 2001). Low SSS may be a more relevant and salient indicator of perceived relative deprivation in young people because they could view it as more consequential than socioeconomic factors at their age (Goodman et al., 2001). Overall, the unique aspects of this developmentally sensitive period, including adolescent goals of independence and neurobiological changes, may render low SSES and low SSS as fundamental threats to an adolescent (Casey et al., 2008; Van der Aar et al., 2018).

Low SSES and SSS may specifically impact food choice, distinct from other behaviors, in adolescents for a few reasons. One explanation is that low SSES may be an evolved cue for adaptation. That is, social and material resources protect organisms from harm, and when an organism perceives that they are relatively deprived of these resources, compensating by acquiring other resources could be key for survival (Nettle et al., 2017). Greater energy intake is among the most conserved survival mechanisms across species; thus, organisms may compensate for low perceived social and material resources by acquiring energy-dense food. In a randomised controlled trial, a low subjective status manipulation increased circulating ghrelin levels, an appetite-stimulating hormone, and blunted sensations of satiation and satiety (Sim et al., 2018); this suggests physiological mechanisms may underlie the compensation. Potential mechanisms may also include increases in impulsivity and sensitivity to food versus other rewards. Low subjective status has been associated with greater desire for immediate rewards (Tunney and Raybould, 2023) and with higher relative reinforcing food, or effort to obtain food versus other rewards (Lin et al., 2013). These physiological and reward mechanisms may become especially active in adolescents due to their rapidly maturing limbic systems concurrent to delayed prefrontal system maturation (Casey et al., 2008).

Non-experimental studies in in children, adolescents, and young adults demonstrate associations of lower self-reported SSES with unhealthful diet quality (Belardinelli et al., 2022), more frequent intake of unhealthy foods (Elgar et al., 2016), less frequent intake of healthy foods (Quon and McGrath, 2015), hyperphagia (Smith et al., 2023), and greater body mass index (BMI; Goodman et al., 2001), though statistical significance of findings is sometimes attenuated when accounting for the influence of objective socioeconomic status (Quon and McGrath, 2014). Non-experimental studies in children, adolescents, and young adults also show associations of lower self-reported SSS with greater intake of unhealthy foods (Rahal et al., 2023), less intake of healthy foods (Rahal et al., 2023), and greater BMI (Goodman et al., 2001; Goodman et al., 2015). Often associations of lower self-reported SSS with these appetite-related outcomes are stronger than those of lower self-reported SSES (Cheon et al., 2024; Goodman et al., 2001; Rahal et al., 2023). For example, in a 15-day ecological momentary assessment, young adults (~age 20 years) who perceived themselves as lower in social status in their college community ate more unhealthy foods and less healthy foods, while their self-reported SSES was not statistically significantly associated with food intake (Rahal et al., 2023).

Experimentally manipulating low SSES in adults has triggered stronger preferences for, and intake of, high-calorie foods across multiple studies (Cheon and Hong, 2017; Cheon et al., 2018). Due to the declines of subjective statuses that occur in adolescence, particularly from ages ≥ 14-years-old (Goodman et al., 2001), and the developmentally sensitive goals and neurobiological changes (Casey et al., 2008; Van der Aar et al., 2018), late adolescence may be a key developmental period during which low SSES and SSS influence food choices. However, no experiments have tested effects of SSES manipulations on appetite-related outcomes in youth younger than 18-years-old, and only one experiment has tested the effects of an SSS manipulation in youth aged 15-21 years (Cardel et al., 2020). Although there was a trend for youth in the low SSS condition to consume greater energy (particularly, from fat), 24-hour energy balance did not statistically significantly differ due to the manipulation (Cardel et al., 2020).

The present research filled literature gaps with the first experiments testing effects of SSES and SSS manipulations on food choice in large samples of American adolescents aged 14-17 years from a range of socioeconomic backgrounds. Experiments were registered with an analysis plan on the Open Science Framework prior to conducting the research (Experiment 1: https://osf.io/y96tv, Experiment 2: https://osf.io/wkftm), and the preregistrations adhered to the disclosure requirements of the institutional registry. Primary aims were to investigate whether SSES and SSS manipulations causally affect the healthfulness of food choices in adolescents ^1^. For Experiment 1, we hypothesized that, compared to the control condition, the low SSES manipulation would cause unhealthier food choices in adolescents. For Experiment 2, we hypothesized that, compared to the control condition, the low SSS manipulation would cause unhealthier food choices in adolescents and the high SSS manipulation would cause healthier food choices. An exploratory aim was to examine associations of self-reported SSES and SSS with healthfulness of food choices, independent of experimental condition and objective socioeconomic status.

## Methods

### Study Designs

Experiment 1 was a 2-level (low SSES or control) randomized between-subjects experiment. Experiment 2 was a 3-level (low SSS, high SSS, or control) randomized between-subjects experiment ^2^.

### Participants

Both experiments involved community samples recruited by Dynata LLC (Planto, Texas). Dynata LLC recruited parents of adolescents aged 14-17 years living in the USA who consented to their child’s participation and received rewards for participating. Dynata LLC targeted these parents, validated their identification with Government Issued IDs, and applied machine learning to conduct initial data checks, improving data quality. For Experiment 1, we sought to recruit 500 adolescents (through parents) based on a power analysis in G*Power Version 3.1.9.6 (Faul et al., 2007) with the following parameters: 2-group between-subjects factors analysis of variance (ANOVA), power = .90, *α* = .05, and a small effect size (Cohen’s *d* = 0.29) as observed in previous research in adults (Cheon and Hong, 2017). For Experiment 2, we sought to recruit 770 adolescents (again, through parents) based on similar approaches as Experiment 1 (≥ 250 participants per condition) and available research funds.

Inclusion criteria were a child 14-17 years old currently living with the parent and parent ≥ 30 years old. For Experiment 1, adolescents had no dietary restrictions and there was a quota for adolescent sex assigned at birth (50% female, 50% male); however, to reduce the number of exclusions, these two criteria were not applied in Experiment 2. In accordance with our preregistration, we conducted an additional data check after Dynata LLC, and data were excluded from analysis if adolescents completed the study in < 3 minutes, displayed haphazard responding (e.g., nonsensical text entry), or reported suspicion of the study purpose. As a part of the SSES/SSS manipulation instructions, adolescents wrote text about an imagined social interaction (see Procedure), and four trained research assistants read through each text response for possible haphazard responding in accordance with our preregistration. In doing so, the research assistants identified responses (*n* = 5) that seemed written by the parents rather than adolescents (e.g., “Would ask about their family and see if they have any children are kind my sons age to play with,” “my family has to budget money carefully so that I am able to pay all of my bills and still have something leftover”). These text responses were reviewed and discussed within the authorship team, and we decided to exclude those participants from analysis.

The final samples were 473 and 775 for Experiments 1 and 2, respectively (see Figure S1 in Supplementary Material for flow charts noting exclusions at each stage). Participants were recruited July 11^th^ to 29^th^ 2022 for Experiment 1 and July 27^th^ to August 3^rd^ 2023 for Experiment 2, with participants from the first experiment unable to participate in the second.

### Procedures

The procedures were approved by the institutional review board of a of a Singaporean university (IRB-2022-076) and were carried out using Qualtrics (Provo, UT). Parents answered questions about family structure and their objective socioeconomic status and, if they met the inclusion criteria, provided informed consent for their adolescent to participate. Adolescents provided assent and rated their baseline hunger on a Visual Analogue Scale. Then, adolescents were randomly assigned to their experimental condition via Qualtrics’ randomizer in survey flow.

The MacArthur Ladder (Adler et al., 2000) is the most common measure of subjective status; individuals are asked to rate their perception of one’s place in the socioeconomic or social structure. Previous experiments testing the effects of SSES on food choice have based manipulations on the MacArthur Ladder (Cheon and Hong, 2017; Cheon et al., 2018) and we took a similar approach here. However, we created the SSES and SSS manipulations based off the youth versions of the MacArthur Ladder (Goodman et al., 2001); these versions were adapted to reflect that adolescents’ SSES may be impacted by their families’ objective socioeconomic status, and their SSS may be most relevant in context of their school communities. For the current research, adolescents viewed the image of a 10-rung MacArthur Ladder (Adler et al., 2000; Goodman et al., 2001) and were provided the following instructions:

**Experiment 1: Low SSES.** “Imagine that the ladder below represents how American society is set up. Now think about your family, and please compare your family to the families **at the very top of the ladder**. These are the families who are the best off—they have the most money, the highest amount of schooling, and the jobs that bring the most respect. We’d like you to think about how **your family is different from these people** in terms of the money, schooling, and jobs that your family has. Where would you place your family on this ladder relative to the families at the very top? … Now imagine that you meet (for the first time) a person your age from a family who is **at the very top of the ladder** in American society. Think about how the **differences between you** might affect (1) what topics you would talk about and (2) how the conversation is likely to go. Please write a brief description (3-5 sentences) about how you think this conversation would go.”
**Experiment 2: Low/high SSS.** “Imagine that the ladder below represents where students stand in your school. Now think about yourself, and please compare yourself to the students **at the very top/bottom of the ladder**. These are the students who are the best/worst off—with the most/least respect, the highest/lowest grades, and the highest/lowest standing. We’d like you to think about how **you are different from these students** in terms of the respect, grades, and popularity that you have. Where would you place yourself on this ladder relative to the students at the very top/bottom?.… Now imagine that you meet (for the first time) a student who is **at the very top/bottom of the ladder** in your school. Think about how the **differences between you** might affect (1) what topics you would talk about and (2) how the conversation is likely to go. Please write a brief description (3-5 sentences) about how you think this conversation would go.”

As a part of these manipulations, adolescents rated themselves on their **first** MacArthur Ladder, ranking their SSES (Experiment 1) or SSS (Experiment 2). In the control conditions, adolescents ranked their SSES (Experiment 1) or SSS (Experiment 2) on the MacArthur Ladder too but after having imagined meeting another adolescent without comparison (see Appendix A in Supplementary Material for full instructions from each condition). Next, all adolescents answered the Multiple Food Test (Schreiber et al., 2020) and questions on demographics. Lastly, all adolescents rated themselves on a **second** MacArthur Ladder, ranking whichever type of subjective status they had not before (self-reported SSS for Experiment 1 and self-reported SSES for Experiment 2); the purpose of this was to have both self-reported SSS and self-reported SSES from adolescents in each experiment.

### Measures

#### MacArthur Ladders

Youth versions of MacArthur Ladders (Adler et al., 2000; Goodman et al., 2001) were used. Adolescents self-reported their statuses from 1 (*bottom rung of ladder*) to 10 (*top rung of ladder*). Higher scores for SSES indicate that adolescents perceived their family as having more money, more schooling, and jobs that bring more respect compared to other American families. Higher scores for SSS indicate that adolescents perceived themselves as having more respect, higher grades, and higher social standing compared to their peers at school.

#### Multiple Food Test

The Multiple Food Test (Schreiber et al., 2020) assessed healthfulness of food choices. Adolescents were shown 18 sets of four images of food items varying in healthfulness (e.g., apple, sweetcorn, fries, chocolate cake) and asked which food they would choose if the foods were offered to them at that moment. The food items were categorized with a score of 1 (*unhealthy*) to 4 (*healthy*) based on nutrition information; a food choice score was created by averaging the healthfulness scores across selected food items. Choice on the Multiple Food Test has predicted real-world food choice in young people (Schreiber et al., 2020).

#### Demographics

Parents reported the education level of the parent in the household with the highest education, from 1 (*no formal schooling*) to 9 (*advanced degree*), and total annual household income without assets in the past year, from 1 (<$20,000) to 15 (≥$150,000).

Adolescents self-reported their age (in years), their sex assigned at birth, whether they were currently dieting to lose weight, and how often they have feeding independence (i.e., how often they eat food they prepared, eat food they have purchased, or choose what they want to eat) from 1 (*almost never*) to 5 (*all of the time*). Disclosures of race/ethnicity are sensitive data and can present as a unique stressor for minority groups (Shaff et al., 2024). We therefore carefully considered recommendations by our institutional review board and social science experts in deciding whether to collect such data from young people. Given the elevated risk of distress in young people, and that testing manipulations or measurement of structural racism and cultural practices was beyond the scope of our aims, we did not ask young people for information on race/ethnicity.

### Data Analysis

The data underlying this article and syntax used in analysis are available via the Open Science Framework: https://osf.io/23gca/. Analysis was conducted in SPSS 29 (IBM Corporation, Armonk, NY). To test hypotheses, one-way ANOVAs were conducted with healthfulness of food choices as the dependent variable and experimental condition as the between-subjects factor. Statistical significance was set at *p* < .05.

## Results

### Descriptive Statistics and Correlations

Table 1 presents analytic sample demographics and descriptive statistics of variables of interest. On average, adolescents were 15 years of age in both studies. Sex assigned at birth was fairly evenly split between male and female, highest parent education levels ranged from less than high school to advanced degree (e.g., MD), and total annual household income ranged from < $20,000 to ≥ $150,000. Table S1 in Supplementary Material presents bivariate correlations among demographics and variables of interest. Consistent with previous research, we observed moderate to large associations among household income, parental education, self-reported SSES, and self-reported SSS (Goodman et al., 2001).

### Randomization Checks

There were no statistically significant differences in age, sex assigned at birth, dieting status, or feeding independence between adolescents across conditions in both experiments (*p*s > .11); however, there were statistically significant differences in baseline hunger [Experiment 1: *F*(1, 471) = 3.90, *p* = .049, *η*^*2*^ = .01, Cohen’s *d* = 0.18, Experiment 2: Omnibus F(2, 772) = 6.30, *p* = .002, *η*^*2*^ = .02, Cohen’s *d* = 0.26]. Including baseline hunger as a covariate in subsequent models did not change the direction, magnitude, or statistical significance of the results (see Appendix B in Supplementary Material for estimates from adjusted models).

### Manipulation Checks

In Experiment 1, adolescents in the low SSES condition self-reported a statistically significantly lower SSES [*M*(*SD*) = 5.69(1.99)] compared to adolescents in the control condition [*M*(*SD*) = 6.04(1.74), *F*(1, 471) = 4.06, *p* = .044, *η*^*2*^ = .01, Cohen’s *d* = 0.19]. The small effect size of the manipulation’s effects on self-reported SSES was comparable to previous research in adults (Cheon and Hong, 2017). In Experiment 2, there were no statistically significant differences in self-reported SSS across conditions [low SSS: *M*(*SD*) = 7.00(1.95), high SSS: *M*(*SD*) = 6.84(1.92), control: *M*(*SD*) = 7.16(1.82), Omnibus *F*(2, 772) = 1.88, *p* = .154, *η*^*2*^ = .01, Cohen’s *d* = 0.14].

### Primary Aims

**Figure 1.**
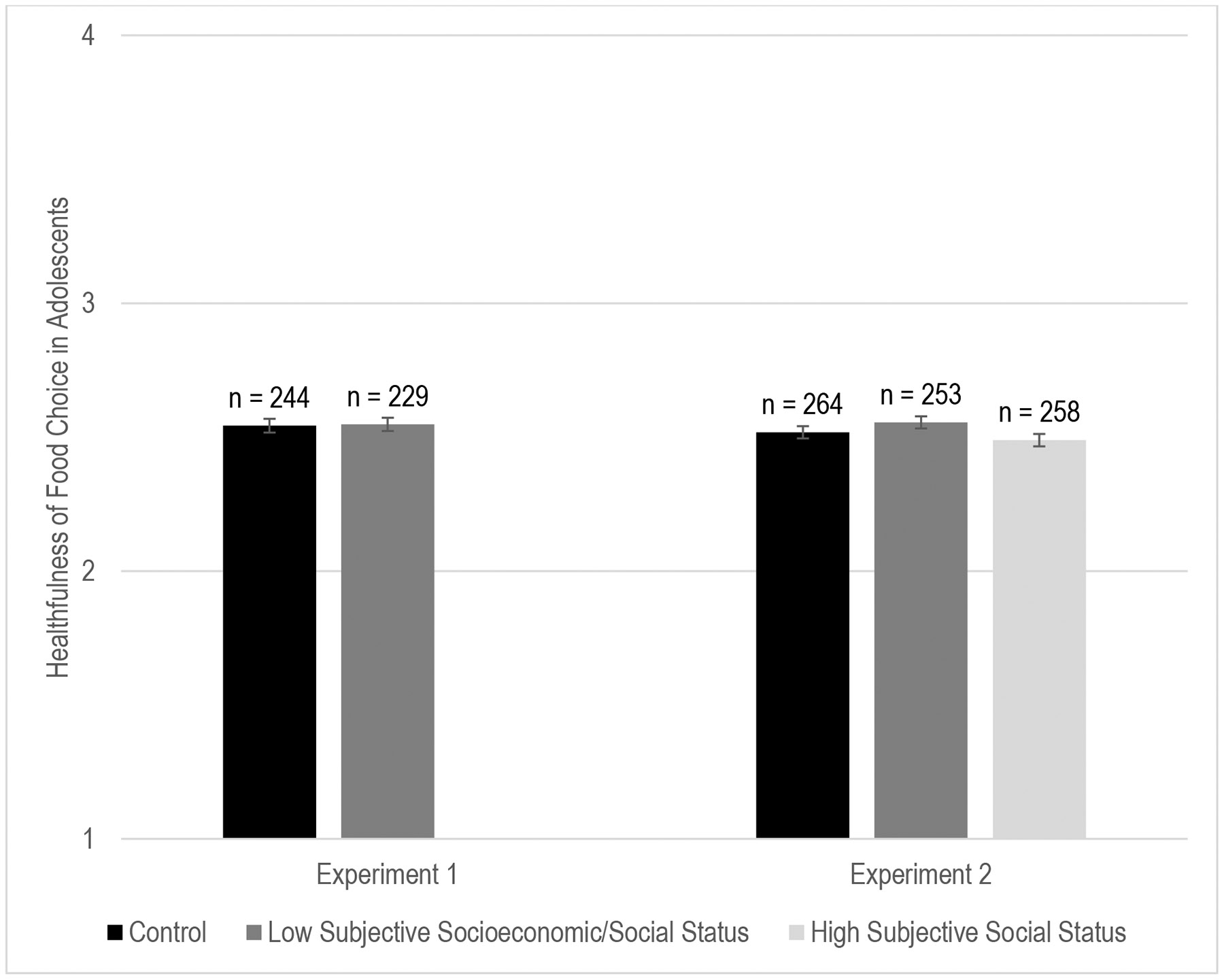
Means and standard errors for the healthfulness of food choices by experimental condition.

As shown in Figure 1, in both experiments, there were no statistically significant differences in the healthfulness of food choices across conditions [Experiment 1: low SSES: *M*(*SD*) = 2.55(0.37), control: *M*(*SD*) = 2.54(0.40), *F*(1, 471) = 0.02, *p* = .889, *η*^*2*^ = .00, Cohen’s *d* = 0.00, Experiment 2: low SSS: *M*(*SD*) = 2.55(0.36), high SSS: *M*(*SD*) = 2.49(0.37), control: *M*(*SD*) = 2.52(0.37), Omnibus *F*(2, 772) = 2.09, *p* = .124, *η*^*2*^ = .01, Cohen’s *d* = 0.14].

### Exploratory Analysis

We examined associations of self-reported SSES and SSS with healthfulness of food choices, independent of experimental condition and objective socioeconomic status (i.e., household income and parental education). Multiple regressions were conducted with variables of interest z-scored (standardized), and conditions dummy coded (Table 1). In Experiments 1 and 2, associations of self-reported SSES with food choice were not statistically significant. However, in Experiment 1, independent of condition and objective socioeconomic status, a 1-SD lower SSS was statistically significantly associated with 0.11-SD (95% CI [0.02, 0.20]) unhealthier food choices. This was replicated in Experiment 2, where independent of condition and objective socioeconomic status, a 1-SD lower SSS was statistically significantly associated with 0.17-SD (95% CI [0.10, 0.25]) unhealthier food choices.

## Discussion

We conducted two preregistered experiments testing effects of subjective status manipulations on food choices in large samples of American adolescents, applying random assignment to create experimental and control groups comparable on background factors including objective socioeconomic status. Although we based our subjective status manipulations on previous experiments (Cheon and Hong, 2017; Cheon et al., 2018), and on youth versions of the MacArthur Ladder (Adler et al., 2000; Goodman et al., 2001), their impacts were very limited in this context. The low SSES manipulation in the first experiment produced a very small effect on self-reported SSES and did not significantly affect adolescents’ food choices. The low and high SSS manipulations in the second experiment did not produce effects on self-reported SSS nor food choices.

In exploratory analysis, adolescents who self-reported lower SSS—that is, adolescents who naturally perceived less respect, worse grades, and worse standings in their schools—chose unhealthier foods independent of any influence from the experimental condition and objective socioeconomic status. This finding was replicated across the two studies and aligns with previous findings that lower self-reported SSS (but not self-reported lower SSES) was associated with greater eating in the absence of hunger among children experiencing teasing distress (Cheon et al., 2024) and greater unhealthy food intake, less healthy food intake, and greater BMI in adolescents and young adults (Goodman et al., 2001; Rahal et al., 2023). Consistent associations of lower self-reported SSS with worse appetite-related outcomes in young people warrants research to determine causality. However, because there were inconsistent effects of the MacArthur Ladder subjective status manipulations on subjective statuses, alternative or refined research methods for testing influences of subjective statuses on food choices in young people are necessary. Below, we discuss methodological considerations from this initial experimental work to guide future research.

First, prior experiments manipulating low SSES were conducted in adults, using a MacArthur Ladder manipulation that focuses on individuals’ places in the socioeconomic structure (rather than their family’s place), and showed that low SSES caused stronger preferences for high-calorie foods and greater energy intake (Cheon and Hong, 2017; Cheon et al., 2018). In our first experiment in adolescents, the very small effect of the low SSES manipulation in decreasing self-reported SSES is important to consider when evaluating the null findings on food choice. Although comparable to previous manipulation effects on self-reported SSES in adults, the effect was quite small. This leaves the possibility that a failure to detect effects on food choice was due to a weak manipulation. Additionally, youth are generally not responsible for acquiring social and material resources for the family and, while emerging in independence, still receive parental support. Due to this, adolescent SSES based on factors independent from their family’s may be more important to manipulate and measure. Another consideration is that participants placed themselves on the MacArthur ladder after comparing themselves with others at the top of the ladder, but before they wrote about differences between themselves and these other peers. In other words, the manipulation check (placement on the MacArthur ladder) was administered before the manipulation was fully completed. Since this was the first experiment testing effects of the MacArthur Ladder SSES manipulation on self-reported SSES and food choice in youth, replication or adaptation of this SSES manipulation is important. The manipulation check could also be administered after (rather than before) adolescents write about differences between themselves and others.

Second, the low SSS and high SSS manipulations using the MacArthur Ladder in our second experiment did not change self-reported SSS nor impact food choice. Again, the participants placed themselves on the MacArthur ladder after comparing themselves with others but before writing about differences, so the manipulation check could be administered later in future research. Another consideration is that asking adolescents to perceive relative deprivation of their household income could have been easier than perceiving relative deprivation of respect, popularity, and achievement because of the salient socioeconomic inequalities in American society (Hirschman et al., 2019). In the only other experiment testing a manipulation of SSS, youth aged 15-21 years played a Monopoly game with a confederate, receiving either the Rolls-Royce piece and starting advantages or the shoe piece and starting disadvantages; although the low SSS condition increased feelings of frustration and decreased feelings of powerfulness, post-manipulation SSS on a MacArthur Ladder was not measured (Cardel et al., 2020). Thus, the existing MacArthur Ladder and Monopoly game manipulations of SSS could be refined or a new approach developed.

Third, it is critical to consider that adolescent subjective statuses, compared to adult subjective statuses, may be more resistant to traditional cognitive manipulations. We focused on adolescence because it is a key period for subjective status to change as individuals begin establishing socioeconomic independence from their parents (Goodman et al., 2001) and neurobiologically become sensitive to social rewards and punishment (Casey et al., 2008). We can only speculate as to why a cognitive manipulation would be ineffective in this developmental context, but perhaps it is because prefrontal systems that underlie higher-order thinking are not fully developed (Casey et al., 2008). This could make it difficult for adolescents to deeply imagine the comparisons they are asked to make; indeed, executive function capacities are related to the ability to think about different perspectives during adolescence (Hollarek and Lee, 2022). Another possibility is that the cognitive manipulation is too brief or artificial compared to longer, real experiences of social comparison in adolescents’ lives (Van der Aar et al., 2018). The instructions are coming from adult researchers rather than other adolescents, which also could make the manipulation less important to young people. Participatory research where adolescents are recruited for a project advisory group and co-design subjective status manipulations is an important next step to expanding this research area. It is also important to consider that SSES and SSS may be difficult to manipulate in youth, so longitudinal studies examining natural fluctuations and subsequent effects may be more informative (Goodman et al., 2015).

Results should be interpreted with regards to study limitations. Although conducting online experiments and recruiting participants via Dynata LLC provided large samples of American adolescents from a range of socioeconomic backgrounds, it increased risks of potentially ineligible participants (e.g., parents) completing the study and of low data quality. These are potential limitations of any online study, since researchers cannot directly observe who is completing the study. We trained four research assistants to read through text responses to the SSES/SSS manipulation instructions, identifying responses that seemed written by the parents rather than adolescents; however, the validity of this method could not be determined. Future research could consider new approaches to reducing risks of false identity like real-time photo verification (Verasight; Livingston, NJ) or recruiting directly from a known adolescent population, such as emailing through schools’ contact lists. The risk of low data quality was mitigated through Dynata LLC’s application of machine learning for data checks, and our preregistered data checks regarding study completion time and haphazard responding or reported suspicion of the study in free text responses. The present study focused on subjective socioeconomic and social factors only within the USA and did not ask adolescents about their race/ethnicity because this is sensitive data that can be stressful for minority groups to disclose (Shaff et al., 2024), and because testing manipulations or measurement of structural racism and cultural practices was beyond our aims. Nevertheless, structural racism or cultural factors may affect subjective status and food choice (Cheon et al., 2022), so future research could weigh the risks and benefits of incorporating these variables and could examine adolescents in other countries.

Another consideration regarding the present research is that separate experiments tested effects of SSES and SSS manipulations on food choice in adolescents. Future research could consider factorial designs that test the interactions between SSES and SSS. It is possible that negative effects of low subjective status in adolescents are most pronounced when they perceive themselves as being lower in both aspects of their social world (Goodman et al., 2001). Furthermore, prior research on the link between subjective status and appetite-related outcomes in adolescents has included dependent measures of diet quality (Belardinelli et al., 2022), frequency of food intake (Elgar et al., 2016; Quon and McGrath, 2015; Rahal et al., 2023), hyperphagia (Smith et al., 2023), BMI (Cheon et al., 2024; Goodman et al., 2001; Goodman et al., 2015), energy intake and 24-hour energy (Cardel et al., 2020), but for the current research, an online food choice task was selected. Although the online food choice task has been validated against offline food choice (Schreiber et al., 2020), enhancing external validity, future research should examine effects of subjective status manipulations on actual food intake of adolescents. Moreover, future research could include a comprehensive set of appetite-related outcomes for comparison within the literature and identification of whether effects are limited to certain types of appetite-related outcomes.

In conclusion, there were inconsistent effects of the MacArthur Ladder subjective status manipulations on subjective statuses and no statistically significant effects on food choices in adolescents. Experimental research with other manipulations of SSES and SSS, or longitudinal research of developmental changes, is needed to test influences on food choice in youth. Understanding how status perceptions impact food intake in youth may reveal insight on the psychological mechanisms by which socioeconomic disparities persist, pointing to novel targets for prevention and intervention. For example, teaching skills for cognitive reappraisals related to SSES and SSS may reduce the potential negative impact of perceiving oneself as lower in status, but further research is needed.

## Supplementary Material

Supplemental Materials

## Figures and Tables

**Table 1:** Demographics of Analytic Samples and Descriptive Statistics for Variables of Interest

	Experiment 1 (*n* = 473)	Experiment 2 (*n* = 775)
	*M(SD) or n(%)*	*M(SD) or n(%)*
Age (years)	15.36 (1.07)	15.42 (1.11)
Sex assigned at birth		
Female	238 (50.3%)	367 (47.4%)
Male	235 (49.7%)	408 (52.6%)
Dieting status		
No	440 (93.0%)	674 (87.0%)
Yes	33 (7.0%)	101 (13.0%)
Independence in feeding (1-5)	3.38 (1.12)	3.40 (1.13)
Hunger at baseline (0-100)	38.01 (26.16)	42.33 (27.94)
Highest parent education level		
No formal schooling	0 (0.0%)	0 (0.0%)
Less than high school	3 (0.6%)	3 (0.4%)
Some high school	14 (3.0%)	10 (1.3%)
High school graduate or GED	97 (20.5%)	100 (12.9%)
Some college but no degree	105 (22.2%)	171 (22.1%)
Associate’s degree	74 (15.6%)	110 (14.2%)
Bachelor’s degree	113 (23.9%)	180 (23.2%)
Master’s degree	56 (11.8%)	170 (21.9%)
Advanced degree (e.g., MD, PhD)	11 (2.3%)	31 (4.0%)
Annual household income		
< $20,000	61 (12.9%)	49 (6.3%)
$20,000-$29,999	55 (11.6%)	47 (6.1%)
$30,000-$39,999	53 (11.2%)	46 (5.9%)
$40,000-$49,999	47 (9.9%)	54 (7.0%)
$50,000-$59,999	42 (8.9%)	60 (7.7%)
$60,000-$69,999	30 (6.3%)	55 (7.1%)
$70,000-$79,999	30 (6.3%)	64 (8.3%)
$80,000-$89,999	17 (3.6%)	44 (5.7%)
$90,000-$99,999	19 (4.0%)	52 (6.7%)
$100,000-$109,999	13 (2.7%)	49 (6.3%)
$110,000-$119,999	6 (1.3%)	23 (3.0%)
$120,000-$129,999	25 (5.3%)	45 (5.8%)
$130,000-$139,999	8 (1.7%)	16 (2.1%)
$140,000-$149,999	23 (4.9%)	44 (5.7%)
≥ $150,000	44 (9.3%)	127 (16.4%)
Subjective socioeconomic status (1-10)	5.87 (1.88)	6.48 (1.85)
Subjective social status (1-10)	6.94 (1.83)	7.00 (1.90)
Healthfulness of food choices (1-4)	2.54 (0.39)	2.52 (0.37)

**Table 2:** Estimates from Multiple Regressions Testing Associations of Subjective Social and Socioeconomic Statuses with Healthfulness of Food Choices in Adolescents

		*b*	*p*	95%CI (Lower, Upper)	*R* ^*2*^
	Healthfulness of food choices				
**Subjective Social Status**	**Experiment 1 (*n* = 473)**				.01
	Condition (0 = control, 1 = low SSES)	0.02	.800	(−0.16, 0.20)	
	Household income	0.00	.991	(−0.11, 0.11)	
	Parental education	−0.08	.171	(−0.18, 0.03)	
	**Subjective social status**	**0.11**	**.022**	**( 0.02, 0.20)**	
	**Experiment 2 (*n* = 775)**				.04
	Condition (0 = control, 1 = low SSS)	0.12	.175	(−0.05, 0.29)	
	Condition (0 = control, 1 = high SSS)	−0.06	.493	(−0.23, 0.11)	
	Household income	−0.07	.110	(−0.16, 0.02)	
	Parental education	−0.04	.437	(−0.12, 0.05)	
	**Subjective social status**	**0.17**	**<.001**	**( 0.10, 0.25)**	
**Subjective** **Socioeconomic** **Status**	**Experiment 1 (*n* = 473)**				.01
	Condition (0 = control, 1 = low SSES)	−0.01	.935	(−0.19, 0.17)	
	Household income	0.05	.365	(−0.06, 0.17)	
	Parental education	−0.05	.331	(−0.16, 0.06)	
	Subjective socioeconomic status	−0.10	.059	(−0.20, 0.00)	
	**Experiment 2 (*n* = 775)**				.01
	Condition (0 = control, 1 = low SSS)	0.10	.268	(−0.08, 0.27)	
	Condition (0 = control, 1 = high SSS)	−0.07	.400	(−0.25, 0.10)	
	Household income	−0.07	.117	(−0.17, 0.02)	
	Parental education	−0.03	.544	(−0.12, 0.06)	
	Subjective socioeconomic status	0.07	.080	(−0.01, 0.15)	

*Notes:* Variables of interest were z-scored (standardized) and conditions were dummy coded. SSES = Subjective socioeconomic status, SSS = Subjective social status

## Data Availability

The data underlying this article and syntax used in analysis are available via the Open Science Framework: https://osf.io/23gca/.

## Footnotes

1 The preregistered analysis plans included tests of mediation that are not presented in this paper but available on the Open Science Framework: https://osf.io/23gca/

2 Experiments manipulating SSES have compared low and high SSES conditions to neutral SSES controls, establishing effects from low (but not high) SSES conditions. Given that the prior experiment manipulating SSS compared only a low to a high SSS condition, we included both low and high SSS conditions and a neutral SSS control. This would allow us to test if effects are from low and high SSS conditions, or only low SSS conditions.
