# Supplementary material for: Effects of Subjective Socioeconomic and Social Statuses on Food Choice in Adolescents
Source: J Health Psychol. Author manuscript; Available in PMC 2026 Jul 1. (PMC13322134; doi:10.1177/13591053261442340)
Supplement: Supplemental Materials [file NIHMS2186224-supplement-Supplemental_Materials.docx]

**Supplementary Material**

**Table S1**

*Bivariate Correlations Among Demographics and Variables of Interest*

| **Experiment 1 (*n* = 473)** | | | | | | | | | | |
| --- | --- | --- | --- | --- | --- | --- | --- | --- | --- | --- |
|  | 1. | 2. | 3. | 4. | 5. | 6. | 7. | 8. | 9. | 10. |
| 1. Age |  |  |  |  |  |  |  |  |  |  |
| 2. Sex assigned at birth | -.06 |  |  |  |  |  |  |  |  |  |
| 3. Dieting status | .00 | -.01 |  |  |  |  |  |  |  |  |
| 4. Independence in feeding | .12* | .05 | -.03 |  |  |  |  |  |  |  |
| 5. Hunger at baseline | -.02 | -.04 | -.03 | .01 |  |  |  |  |  |  |
| 6. Highest parent education level | -.03 | -.05 | .00 | .04 | .06 |  |  |  |  |  |
| 7. Annual household income | -.01 | -.08 | -.04 | -.01 | .05 | .55*** |  |  |  |  |
| 8. Subjective socioeconomic status | .01 | .00 | -.07 | .07 | -.04 | .28*** | .40*** |  |  |  |
| 9. Subjective social status | .05 | -.01 | -.01 | .14** | -.06 | .23*** | .24*** | .62*** |  |  |
| 10. Healthfulness of food choices | .03 | .07 | .01 | .07 | -.07 | -.05 | -.02 | -.09 | .09* |  |
| **Experiment 2 (*n* = 775)** | | | | | | | | | | |
|  | 1. | 2. | 3. | 4. | 5. | 6. | 7. | 8. | 9. | 10. |
| 1. Age |  |  |  |  |  |  |  |  |  |  |
| 2. Sex assigned at birth | -.05 |  |  |  |  |  |  |  |  |  |
| 3. Dieting status | .02 | .06 |  |  |  |  |  |  |  |  |
| 4. Independence in feeding | .10** | .06 | .03 |  |  |  |  |  |  |  |
| 5. Hunger at baseline | .05 | .00 | .08* | .01 |  |  |  |  |  |  |
| 6. Highest parent education level | .01 | -.04 | .09* | -.04 | -.02 |  |  |  |  |  |
| 7. Annual household income | .04 | -.04 | .02 | .00 | -.05 | .61*** |  |  |  |  |
| 8. Subjective socioeconomic status | .07 | -.06 | .01 | .01 | .01 | .30*** | .38*** |  |  |  |
| 9. Subjective social status | .03 | -.05 | -.04 | .05 | .03 | .16*** | .17*** | .44*** |  |  |
| 10. Healthfulness of food choices | .07 | .07 | .04 | .10** | -.07 | -.05 | -.06 | .04 | .16*** |  |

*Notes:* Sex assigned at birth dummy coded (0 = Male, 1 = Female). Dieting status dummy coded (0 = No, 1 = Yes). ****p* < .001, ***p* < .01, **p* < .05

**Figure S1**

*Flow Chart Indicating Participant Exclusions at Each Stage*


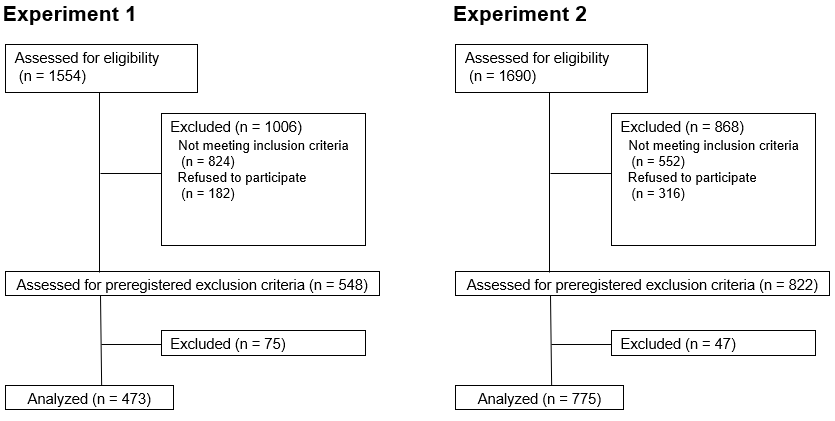


**Appendix A**

**Experiment 1: Low subjective socioeconomic status.** “Imagine that the ladder below represents how American society is set up. Now think about your family, and please compare your family to the families **at the very top of the ladder**. These are the families who are the best off—they have the most money, the highest amount of schooling, and the jobs that bring the most respect. We’d like you to think about how **your family is different from these people** in terms of the money, schooling, and jobs that your family has. Where would you place your family on this ladder relative to the families at the very top? Please note the numbers beside each rung of the ladder. A 10 represents the top rung of the ladder and a 1 represents the bottom rung of the ladder. Please select the number that corresponds to the rung where you think your family stands in relation to the families at the very top… Now imagine that you meet (for the first time) a person your age from a family who is **at the very top of the ladder** in American society. Think about how the **differences between you** might affect (1) what topics you would talk about and (2) how the conversation is likely to go. Please write a brief description (3-5 sentences) about how you think this conversation would go.”

**Experiment 1: Control condition.** “Imagine that the ladder below represents how American society is set up. At the top of the ladder are the families who are the best off— they have the most money, the highest amount of schooling, and the jobs that bring the most respect. At the bottom are the families who are the worst off—they have the least money, little or no education, no jobs or jobs that no one wants or respects. Now think about your family. Where would you place your family on this ladder? Please note the numbers beside each rung of the ladder. A 10 represents the top rung of the ladder and a 1 represents the bottom rung of the ladder. Please select the number that corresponds to the rung where you think your family would be on this ladder… Now imagine that you meet (for the first time) a person your age from a family in American society. Please write a brief description (3-5 sentences) about what topics you would talk about and how the conversation is likely to go.”

**Experiment 2: Low subjective social status.** “Imagine that the ladder below represents where students stand in your school. Now think about yourself, and please compare yourself to the students **at the very top of the ladder**. These are the students who are the best off—with the most respect, the highest grades, and the highest standing. We’d like you to think about how **you are different from these students** in terms of the respect, grades, and popularity that you have. Where would you place yourself on this ladder relative to the students at the very top? Please note the numbers beside each rung of the ladder. A 10 represents the top rung of the ladder and a 1 represents the bottom rung of the ladder. Please select the number that corresponds to the rung where you think you stand in relation to the students at the very top.… Now imagine that you meet (for the first time) a student who is **at the very top of the ladder** in your school. Think about how the **differences between you** might affect (1) what topics you would talk about and (2) how the conversation is likely to go. Please write a brief description (3-5 sentences) about how you think this conversation would go.”

**Experiment 2: High subjective social status.** “Imagine that the ladder below represents where students stand in your school. Now think about yourself, and please compare yourself to the students **at the very bottom of the ladder**. These are the students who are the worst off—with the least respect, the lowest grades, and the lowest standing. We’d like you to think about how **you are different from these students** in terms of the respect, grades, and popularity that you have. Where would you place yourself on this ladder relative to the students at the very bottom? Please note the numbers beside each rung of the ladder. A 10 represents the top rung of the ladder and a 1 represents the bottom rung of the ladder. Please select the number that corresponds to the rung where you think you stand in relation to the students at the very bottom… Now imagine that you meet (for the first time) a student who is **at the very bottom of the ladder** in your school. Think about how the **differences between you** might affect (1) what topics you would talk about and (2) how the conversation is likely to go. Please write a brief description (3-5 sentences) about how you think this conversation would go.”

**Experiment 2: Control condition.** “Imagine that the ladder below represents where students stand in your school. At the top of the ladder are the students in your school who are the best off—with the most respect, the highest grades, and the highest standing. At the bottom are the students in your school who are the worst off—whom no one respects, whom no one wants to hang around with, and who have the worst grades. Now think about yourself. Where would you place yourself on this ladder? Please note the numbers beside each rung of the ladder. A 10 represents the top rung of the ladder and a 1 represents the bottom rung of the ladder. Please select the number that corresponds to the rung where you think you stand in relation to students at your school… Now imagine that you meet (for the first time) a student from your school. Please write a brief description (3-5 sentences) about what topics you would talk about and how the conversation is likely to go.”

**Appendix B**

**Manipulation Checks**

In Experiment 1, when controlling for baseline hunger, adolescents in the low subjective socioeconomic status condition reported a statistically significantly lower subjective socioeconomic status [*M*(*SE*) = 5.69(0.12)] compared to adolescents in the control condition [*M*(*SE*) = 6.05(0.12), *F*(1, 470) = 4.44, *p* = .036, *η^2^* = .01, Cohen’s *d* = 0.19]. In Experiment 2, when controlling for baseline hunger, there were no statistically significant differences in subjective social status across experimental conditions [Omnibus *F*(2, 771) = 1.79, *p* = .167, *η^2^* = .01, Cohen’s *d* = 0.14].

**Primary Aims**

In both experiments, when controlling for baseline hunger, there were no statistically significant differences in the healthfulness of food choices across conditions [Experiment 1: *F*(1, 470) = 0.00, *p* = .997, *η^2^* = .00, Cohen’s *d* = 0.00, Experiment 2: Omnibus *F*(2, 771) = 2.65, *p* = .071, *η^2^* = .01, Cohen’s *d* = 0.17].
